# Subjective refraction and prescribing styles used by UK optometrists

**DOI:** 10.1111/opo.13495

**Published:** 2025-04-03

**Authors:** Jeremy Beesley, Christopher J. Davey, David B. Elliott

**Affiliations:** ^1^ Bradford School of Optometry and Vision Science University of Bradford Bradford UK; ^2^ Present address: Specsavers Beverley UK

**Keywords:** autorefractor, cylinders, prescribing maxims, subjective refraction, visual acuity

## Abstract

**Purpose:**

To investigate the methods of subjective refraction and prescribing used by UK optometrists in routine eye examinations.

**Methods:**

Following a pilot study of 12 observed refractions conducted by nine optometrists, a questionnaire consisting of simple questions regarding methods used together with conditional response clinical vignettes was constructed. Paper copies were distributed to UK optometrists attending continuing professional development (CPD) courses, in addition to an online version.

**Results:**

Two hundred and four completed questionnaires were gathered from respondents with a mean experience of 16 years (SD, 12 years). Poor technique was defined as likely to contribute to or cause a recheck according to a previous study. Poor techniques seen included visual acuity (VA) not being measured to a full threshold level by 93% of respondents, 37% (55/149) prescribing a small oblique cylinder to an asymptomatic patient and 39% (71/183) making just one subjective change to the axis of a −1.00 D cylinder from their starting prescription before prescribing that axis change to a mildly symptomatic patient. The risk of over‐plusing was identified, with 28% (*N* = 46) of respondents happy to prescribe a full objective +0.50 DS change to a patient with no distance‐related symptoms. Symptoms, prescription change and VAs are often not reconciled, with 43% (82/189) of respondents reporting being happy to prescribe a full subjective change of −2.00 DS together with −0.75 DC for a 72‐year‐old patient for a relatively small VA improvement of 6/15 to 6/7.5.

**Conclusions:**

Poor subjective refraction and prescribing techniques were identified; limited measurement of VA to full threshold, risk of over‐plusing/under‐minusing, limited cylinder power and axis techniques and symptoms often not reconciling with changes in prescription and improvements in VA were observed. The objective prescription, particularly as determined by autorefractors, holds an exaggerated influence over some optometrists. These are elements of everyday optometry, yet require greater emphasis in CPD.

**Key points**

- Poor refraction and prescribing techniques by some UK optometrists were identified using a questionnaire of simple questions and clinical vignettes.
- Poor technique with refraction and prescribing is likely to increase the risk of patients' non‐acceptance of a new spectacle prescription.
- Greater emphasis on refraction and prescribing in optometrists' continuing professional development is required.

## INTRODUCTION

A recent study^1^ identified the principal causes of patient complaints with new spectacles, with 83% of prescription‐related rechecks assessed as errors of refraction/prescribing and just 17% attributed to a failure of patient adaptation to an accurate prescription. In a follow‐up continuing professional development (CPD) paper,^2^ recommendations regarding prescribing were made: consideration of presenting symptoms and reconciling these with changes in prescription and improvements in visual acuity (VA), risks of over‐plusing/under‐minusing and care with cylinder changes, particularly oblique cylinders. Assessment of the causes of the rechecks suggested that some elements of refraction are sometimes missing, with other elements performed in a suboptimal way, for example, inappropriate (solely binocular) or missing measurement of habitual VA, preventing reconciling with symptoms and prescription changes.^1^, ^2^

Previous studies have investigated prescribing approaches used by UK optometrists to attempt to avoid rechecks^3^, ^4^; however, what is not currently well understood are the techniques used in conducting the subjective refraction element of a routine eye examination, and there appears to be minimal research evidence for what constitutes a gold standard subjective refraction technique. Discussions with supervisors at university clinics suggest that the teaching of subjective refraction methods can vary significantly with some strongly held views that other clinicians disagree with (e.g., use of ±0.50 or ±0.25 DS confirmation lenses, or how the maxim ‘maximum plus for maximum visual acuity’ [MPMVA]^5^, ^6^, ^7^, ^8^ should be applied). Post‐qualification exchanges of ideas with colleagues together with personal experience of techniques that appeared to work or not work with some patients will likely add to the variety. If some techniques or styles of subjective refraction are suboptimal, the risk of an error of refraction likely increases with consequent increase in the likelihood of patient problems with the new prescription.

Refraction and prescribing are strongly linked. Some experienced optometrists use prescribing maxims/rules to modify the prescribed refractive correction for select patients, typically partially prescribing the change in correction suggested by the refraction to limit the difference to the habitual correction.^3^, ^4^, ^9^ This helps some patients to better adapt to the new spectacles.^3^, ^4^, ^9^, ^10^ Other optometrists curtail the refraction when they have reached the largest change from the habitual correction they would prescribe, and we have termed this a partial refraction.^11^ However, the limitation of the latter approach is that the full refractive correction is not determined, so the optimal VA is not determined either, and only 5% or less^11^ of optometrists reported using this technique even for patients where it may have been suitable. Given this strong link between refraction and prescribing, we have included some aspects of prescribing within the questionnaire.

The aim of this study was to investigate the techniques or styles employed in conducting a routine subjective refraction by UK optometrists, particularly those techniques that might increase the risk of patient complaints with new spectacles, specifically, techniques that might lead to inaccurate cylinder changes or increased risk of over‐plusing/under‐minusing, or cause issues with near working distance, which were the three greatest causes of refraction errors in the recheck study^1^ at 38%, 26% and 12%, respectively. Additionally, the aim was to investigate the extent to which symptoms are reconciled with the link between prescription changes and VA improvements, which is an important aspect of linking refraction to prescribing.^1^, ^2^

## METHODS

Ethical approval was obtained from the University of Bradford Ethics Committee (E977). A pilot study was conducted with the aim of highlighting different styles of refraction, with a particular note made of those styles that might be considered to increase the risk of a recheck or could be considered suboptimal in comparison with standard UK university‐taught methods. Twelve eye examinations by nine optometrists were observed and recorded on a digital sound recorder, with notes made of the subjective refraction element. The nine optometrists, 1–34 years of experience, were from three different practices and included independent, locum and multiple‐group clinicians. Three optometrists had two eye examinations recorded in order to give an estimate of intra‐clinician variability in addition to inter‐clinician variability. The pilot eye examinations suggested that a large variety of techniques were being used. Some (4/12) appeared to strongly ‘push the plus’ (MPMVA) and there appeared to be possible over‐reliance on an autorefractor result (3/12), with symptoms seldom reconciled with prescription changes. Many (9/12) made no effort to check and measure the habitual reading distance or to assess the range of clear vision for near. It became clear during the pilot study that the nuances of changes of lens during the refraction could not be followed by observation, either by a standardised patient or a third party. With consideration of the pilot study results and the errors in refraction suggested by the recheck study,^1^ a questionnaire using clinical vignettes and direct questions was constructed inviting anonymous completion by UK optometrists, with a completion time of <10 min. To minimise the potential bias that clinicians might report more of what they consider would be expected than what they actually do,^12^ the vignettes were constructed to appear as cases an optometrist might routinely encounter in practice and respondents were invited to imagine they were refracting such a patient. To further reduce potential respondent bias, some questions were constructed with a filter, or conditional branching format, where each part of the question was determined by the response to the previous part. This aimed at encouraging respondents to make a flow of decisions, as they would in practice. Space was provided with most questions as an opportunity for respondents to add extra detail that they might feel would better explain what they do. For example, if asked whether they would check for improvements in VA before increasing the cylinder power, most might answer in the affirmative. They were therefore not asked this specific question, but space was provided for them to add such detail if it reflected what they would do. Based on errors made in the recheck study, questions targeted risk of over‐plusing, cylinder axis measurement, cylinder power measurement, determining and prescribing a small oblique cylinder (−0.25 × 45), a large minus and cylinder increase for the improvements in VA, a large reduction in hyperopia and increase in cylinder power with associated increase in anisometropia for an elderly patient and the methods used to determine a reading addition.^1^, ^2^ The questionnaire was given to six optometrists (1–9 years of experience) to complete, and feedback suggested no difficulties regarding clarity of the question. The completion time was in the order of 10 min. The questionnaire is provided in Appendix 1.

Distribution was by paper copies to colleague optometrists plus those attending CPD courses, and by online copies using Google Forms with the link https://tinyurl.com/refmethod and advertisements through WhatsApp, email and *Optician* magazine (ISSN 0030.3968). Data collection was conducted from October 2022 to March 2023, with the questionnaire results including any comments, transcribed onto an Excel spreadsheet for analysis.

With a population of >14,000 optometrists,^13^ for 95% confidence, ±5% margin of error and a response distribution of <15% (we anticipated a majority of respondents would likely adopt established methods as taught in UK university optometry departments), a sample of 194 returned questionnaires was targeted.^14^, ^15^ As the target population would likely have an active interest in the subject, it was anticipated that this target would be achieved.^16^

## RESULTS

### Summary

Two hundred and four questionnaires were returned: 125 paper copies and 79 electronic. Some were incomplete, and the percentages quoted were the proportion of completed responses for each question. Years qualified had a skewness of 0.40 and kurtosis of −0.89, suggesting an approximately normal distribution.

### Autorefractor versus retinoscope users

Users of an autorefractor were more likely to start the subjective refraction with the objective result than retinoscope users (*χ* ^2^ = 4.2, *p* = 0.04) (Questions 5–9) and were much more likely to prescribe the full objective result than retinoscope users (*χ* ^2^ = 16.8, *p* < 0.001) (Questions 6–10). Ninety‐four percent (137/146) of autorefractor users worked in multiple practices.

### Reliance on the objective prescription

Questions 7 and 8 represent asymptomatic patients whose objective refractions have a change in cylinder power and a small oblique cylinder compared to the habitual correction. Fourteen percent (16/115) indicated that they would prescribe the objective result for both asymptomatic patients, where 115 is the number of complete responses for both questions.

Questions 9 and 10 describe patients who have symptoms, with a large objective change for those symptoms and a change in VA. Fifteen percent (27/180) indicated that they would prescribe the objective result for both patients 9 and 10, where 180 is the number of complete responses for both questions.

### Reconciling changes of prescription with symptoms and VA

From Question 9, 24% (44/182) indicated that they would prescribe the full objective change of an increase of −0.75 DS and the full change of −0.75 DC, 10° towards the oblique meridian.

From Question 10, 43% (82/189) indicated that they would prescribe the full subjective change of −2.00/−0.75 × 180 without performing any checks (checks suggested by some respondents included +1.00 blur *N* = 6, duochrome *N* = 1 and binocular balancing/check for binocularity *N* = 3).

### Those who might stop a subjective refraction at a point where a modified prescription would be issued (part refracting)

From Question 7 (a −0.50 DC increase in objective prescription for an asymptomatic patient), 24% (34/141) indicated that they would leave the prescription at −0.50 DC or leave at −0.75 DC without further changes, where 141 is the number of complete responses to this part of the question.

From Question 5 (a +0.50 DS objective increase in a hyperopic prescription), eight respondents indicated that they would leave the prescription at their starting prescription, without changes (*N* = 4 for each: leaving at +3.00 DS, the habitual or leaving at +3.25 DS).

From Question 6 (an objective 20° change of a −1.00 D cylinder towards the oblique), five respondents indicated that they would leave the axis at the habitual axis without any checks.

From Question 8 (an objective −0.25 × 45 cylinder), five respondents indicated that they would not start with the cylinder and would not check for it either.

Twenty‐three percent (47/204) indicated that they might part refract any one or more scenarios. Just four would part refract more than one scenario, one would part refract three scenarios and none would part refract four.

### Risk of overcorrecting the cylinder power

From Question 7 (a −0.50 DC objective increase), 35% (43/123^†^) indicated that they would prescribe a full −0.50 DC change to an asymptomatic patient.

From Question 8 (an objective −0.25 × 45 cylinder), 37% (55/149^†^) indicated that they would prescribe a small oblique cylinder to an asymptomatic patient.

From Question 9 (a large objective change of −0.75 DS and −0.75 DC axis 35° to 45° for the improvement in VA), 23% (44/182^†^) indicated that they would prescribe a full −0.75 DC oblique change.

From Question 10 (a large unilateral prescription change of −1.75/−0.75 × 180), 52% (98/189^†^) indicated that they would prescribe a full −0.75 DC change for an elderly patient.

Twenty‐two percent (45/204) indicated that they would prescribe in two of the four questions 7–10, 10% (21/204) in three of the four and 1% (3/204) in all four. [^†^In this section, the denominator indicates the number of complete responses for the question.]

### Risk of overcorrecting the cylinder axis

From Question 6 (a change of cylinder axis towards the oblique), 39% (71/183) indicated that they would make just one change to the axis from their starting prescription before prescribing that axis. Out of these 71 respondents, 51(72%) used an autorefractor versus a retinoscope.

Further, 14/183 (8%) would leave the axis at the full change of 20° and 41/183 (22%) would leave the axis at a change of 10°–15° after making just one change from their starting prescription. The mean year of experience of these 71 respondents was 16 years.

From Question 9 (a large objective prescription change for the improvement in VA), 55% (100/182) indicated that they would prescribe −1.00 or −1.25 DC, full 10° oblique change.

### Risk of over‐plusing

From Question 5 (a +0.50 DS objective increase in a hyperopic prescription), 28% (46/164) indicated that they would start with +3.50 DS (the objective result) and prescribe this. Of these 46, one suggested that they would check with a duochrome and one that they would check with visual targets outside the test room.

From Question 5, 12% (19/164) indicated that they would start with +3.25, use a +0.25 DS confirmation lens and increase by 0.25 DS. Note, the 19 respondents does not include those who would start with +3.00 DS (51%, 88/164), would increase to +3.25 DS and then increase further to +3.50 DS. This number is unknown, as is the number who would then prescribe +3.50 DS.

From Question 5, of the 27 who report using a +0.50 DS confirmation lens (and either −0.25 or −0.50 DS) and who would start their subjective tests with either +3.00 or +3.25 DS, 24 (15% of 164 total responses) would change the prescription by 0.25 DS and just three by the full 0.50 DS.

### Risk of overcorrecting (over‐minusing)

From Question 9 (a large objective prescription change for the improvement in VA), 24% (44/182) indicated that they would prescribe the full objective change (−1.12 D) for the improvement in VA from 6/9^+2^ to ‘bottom‐line VA’.

### Over‐increase of anisometropia

From Question 10 (an elderly patient with a large unilateral prescription change), 43% (82/189) indicated that they would prescribe the full subjective prescription without performing any checks.

### Reading addition determination (Question 11)

Thirty‐two percent (59/184) did not report that they would confirm the habitual near distance.

Seventy‐two percent (132/184) did not report that they would check the range of clear vision at near.

### Summary of respondents who would start the subjective refraction with the objective prescription versus the habitual prescription

Combining Questions 5, 6, 7, 8 and 9, 7% (14/204) stated that they would start with the objective result for all questions. Fourteen percent (29/204) stated that they would start with the habitual prescription for all questions.

### Completion rates for individual questions

See Table 1.

**TABLE 1:** Question (Q) completion rates (percentages are a proportion of 204 respondents).

	Q5	Q6	Q7	Q8	Q9	Q10	Q11
Incomplete answers, *N*	28	18	64^a^	6	22	15	20
Answered all parts	12	3	17	49			
Total *N* complete responses^b^	164	183	123	149	182	189	184
	80%	90%	60%	73%	89%	93%	90%

^a^ 46/64 were no answers to the last part, whether to keep at −1.00 DC or to reduce to −0.75 DC.

^b^ Complete responses to all parts of the question. Questions 5, 6, 7 and 8 were conditional questions that required the respondent to follow a train of thought, rather than provide a single, brief answer. Just 26 (13%) respondents offered a comment in question 12, with most simply reflecting their answers to previous questions. Some more general comments included ‘would consider the quality of patient responses’, ‘start with the habitual as like to see how VA improves’, ‘much more an art than people realise’ and ‘found some questions confusing’.

## DISCUSSION

This study investigated the approaches towards subjective refraction and prescribing for patients with good VA and reliable subjective responses, as reported by UK optometrists, a population of >14,000.^17^ Our sample size calculation assumed a response distribution of <15%, which is lower than the typical default setting of 50%, which is the most conservative assumption.^14^, ^15^ If the 50% response distribution was a better assumption of the responses gained (we do not believe that this is the case as responses should not be completely random), then our sample of 204 respondents would give a margin of error of ±7%,^15^ that is, if a particular answer was given by 75% of participants, a ±7% margin of error would suggest that the true response is 68%–82% rather than 70%–80% for a ±5% margin of error. The true margins of error may be between these two ranges, and the differences appear minimal. The respondent demographics (Table 2) appear broadly representative compared to data from a survey of the profession conducted by the General Optical Council^17^ in way of gender (equal at 43% male), employed (83% vs. 80%) or working as a locum (17% vs. 20%), although there were more respondents working in multiple practice (68% vs. 51%) and fewer in independent practice (25% vs. 35%). One of the distribution CPD courses was organised by a large multiple, and so while independent and locum clinicians were able to attend, the majority from this distribution method would have belonged to the multiple group.

**TABLE 2:** Respondent demographics and refraction equipment routinely used, % (*N*).

Male	43% (87)
Female	57% (116)
Years qualified, mean	16 years (SD, 12 years)
Male mean years qualified	19 years (SD, 13 years)
Female mean years qualified	14 years (SD, 11 years)
Independent clinician	25% (54)
Multiple clinician	68% (146)
Hospital	4% (8)
University	2% (5)
>1 practice type	(11)
Employed	83% (172)
Locum	17% (36)
Both locum and employed	(6)
*Refraction equipment*	
Trial frame and lenses	41% (95)
Phoropter	59% (137)
Both trial frame and phoropter	(28)
Retinoscope	34% (74)
Autorefractor	66% (146)
Both retinoscope and autorefractor	(16)
Retinoscope users, mean years qualified	25 years (SD, 12 years)
Autorefractor, mean years qualified	13 years (SD, 11 years)

*Note*: No answers: Gender, *N* = 1; practice type, *N* = 2; employed/locum, *N* = 2. There was no significant difference between male and female for type of practice (*χ* ^2^ = 4.3, *p* = 0.23).

### Question type

A possible weakness of a questionnaire‐based study is that respondents might not report what they actually do in practice.^12^ The conditional response questions perhaps have the advantage of better representation of the thought processes applied in practice, together with providing a method of investigating nuances of technique. However, the number of complete responses was slightly lower for this type of question (Table 1), with some respondents answering all parts of the question, showing either a lack of understanding or incomplete reading of the question. We feel that the investigation of nuances of technique that this type of question permits outweighs the disadvantage of lower completion rates. Completion rates of about 90% for Questions 9, 10 and 11 suggest that the questionnaire had a slightly overlong completion time. In the construction of similar questionnaires, the use of longer, conditional response questions should be balanced against question complexity and the length of the questionnaire.

### Poor VA measurements

The mean VA of a normal, healthy 20‐ to 30‐year‐old with optimal refractive correction is approximately −0.14 logMAR or 6/4.5^+1^ Snellen, with an upper 95% confidence limit of −0.26 logMAR or 6/3^−2^ Snellen.^18^ Therefore, if the smallest line of VA used is 6/4.5 (Question 3), the VA improvement for a small change in prescription (e.g., −0.25 DC) would be missed for half of these patients. Measuring VA with a ‘bottom line’ of 6/4.5 will not measure a threshold for many young patients, and at least 93% of respondents do not measure VA to threshold for young patients (Table 3). Indeed, given that 6/6 is the mean VA for 70‐year‐old patients with healthy eyes,^18^ 22% of clinicians do not measure VA to threshold for older patients. Habitual VA appears to be either not measured at all, measured binocularly or measured inaccurately.^1^ Clinicians should be encouraged through CPD to measure habitual monocular visions and/or VA in all patients, to use test charts that have a line smaller than 6/4.5 and to record acuities to a full threshold level. The recording of either visions and/or habitual VA will likely depend on whether the patient wears spectacles full‐time, part‐time or not at all; however, habitual VAs are essential in patients who wear spectacles in order to consider the benefits of any proposed change in prescription.

**TABLE 3:** Questions 1–4.

Question		% (*N*)^a^
(1) When determining the sphere power, which confirmation lenses do you routinely use?	+0.25 and −0.25 DS	75% (154)
	+0.50 and −0.25 DS	12% (25)
	+0.50 and −0.50 DS	13% (27)
	Both ±0.25 and +0.50/−0.25 DS	(4)
(2) For cylinder axis and power, which cross‐cylinder do you routinely use?	±0.25 DC	73% (149)
	±0.50 DC	27% (54)^b^
(3) What is the bottom line you routinely use to measure visual acuity in young adults (20–30 years of age)?	6/7.5	(1)
	6/6	22% (45)
	6/5	46% (93)
	6/4.8	(1)
	6/4.5	23% (47)
	6/4	3% (6)
	6/3.8	3% (7)
	6/3	(1)
(4) At university, were you taught to ‘push the plus’?	Yes	91% (185)
	No	3% (6)
	Cannot remember	6% (12)

^a^ No answers: Question 1 (2), Question 2 (1), Question 3 (3) and Question 4 (1).

^b^ Of the 54 who used ±0.50 DC, 33 (61%) used ±0.25 DS confirmation lenses.

In practice, optometrists may be refracting depending on patient responses, changing the prescription according to a response on an above‐threshold line and simply recording the VA at the end of the refraction. A smallest line used of 6/6 may therefore be considered satisfactory by some, as the patient reports seeing this line more or less clearly with small changes in prescription. Reasons for above‐threshold measurement could include time‐saving or not wishing to leave a patient having ‘failed’ to see the ‘bottom line’. However, we believe that this is flawed as if an incremental change in prescription results in a decrease in VA from 6/3.8 to 6/4.5; for example, the impact of such a change might not be noticed (e.g., the response to a +0.25 DS lens might be that the letters look ‘the same’), with the associated risk of over‐ or under‐correcting.

### Use of ±0.50 D confirmation and cross‐cylinder lenses

Prescriptions following a recheck have been shown to differ from the non‐tolerated prescription by just ±0.50 D or less in 84%^19^–86%^1^ of cases. Therefore, as prescription changes as small as ±0.25 DS, or particularly an oblique ±0.25 DC, can cause problems, rechecks could be caused with small errors of measurement. Clinicians who use ±0.50 DS confirmation lenses (13%, Table 3) or a ±0.50 DC cross‐cylinder (27%, Table 3) with patients who have good VA and provide good subjective responses are at particular risk of inaccurate prescription determination if these are not applied carefully, although confirmation lenses and cross‐cylinders of ±0.50 D or even ±1.00 D may be appropriate with some patients, such as those with low vision. For patients with good vision, the use of +0.50 DS together with increases of +0.25 DS might be considered a good strategy and not likely to over‐correct, and this was used by 24/27 respondents (Question 5). (This method is also used by author JB, with associated bias towards this approach). Where a +0.25 DS confirmation lens is used together with increases of 0.25 D without further checks (and taking no account of the 0.17 D dioptric length of a 6‐m test distance) the patient could be at risk of being over‐plused. There were 19/164 (12%) respondents who would start with +3.25 DS and increase to +3.50 DS, and none of them suggested that they would check with the VA. Added to these are respondents who would start with +3.00 DS, increase to +3.25 DS and then further to +3.50 DS, but this number is unknown.

Although the use of a ±0.50 DC cross‐cylinder might be appropriate with patients who do not or cannot provide accurate responses,^8^ it could cause inaccuracies if not carefully applied, particularly for small powers of 0.25 or 0.50 D (Appendix 2), and so it was surprising that 27% (*N* = 54) respondents reported its routine use. Interestingly, of the 27% (54/203) respondents who reported routine use of a ±0.50 DC cross‐cylinder, 33 (61%) use ±0.25 DS confirmation lenses. It appears that these respondents are more concerned with sphere accuracy than with that of cylinder.

A recent study compared the use of 0.05 versus 0.25 D intervals of prescription in two groups of pre‐presbyopic myopes.^20^ The authors concluded that refractions performed and spectacles glazed to 0.05 D increments gave better visual performance (were preferred by most participants) compared with 0.25 D. Refractions in the future might use smaller steps than 0.25 D for some patients, and consequently, the use of 0.50 DS confirmation lenses and 0.50 DC cross‐cylinders might become even less acceptable for patients with good VA and reliable subjective responses.

### Over‐reliance on autorefractor results

There appears to be an over‐reliance on the objective result by some clinicians, with 14% happy to prescribe the full objective prescription for a patient with cylinder changes but who is asymptomatic (Questions 7 and 8) and 15% happy to prescribe the full objective result for patients with symptoms but large objective changes for the improvements in VA (Questions 9 and 10). Users of an autorefractor were much more likely to prescribe the full objective prescription than retinoscope users (combining Questions 6–10, *χ* ^2^ = 16.8, *p* < 0.001), indicating an over‐reliance on the autorefractor, as previously suggested.^1^, ^2^ Additionally, the influence of an autorefractor on technique is evidenced by the 72% (51/71) of those who make just one change to the cylinder axis before prescribing (Question 6) who use an autorefractor for the objective prescription determination (*χ* ^2^ = 6.68, *p* = 0.01). Users of autorefractors were less experienced than retinoscope users (mean 13 vs. 25 years, two‐tailed *t*‐test, *p* < 0.001), and so this over‐reliance on the objective result could be a function of experience. As 94% of autorefractor users worked in multiple practice, it could also be a function of other constraints (e.g., time) placed upon clinicians by the type of practice.

When refracting and prescribing for patients with communication difficulties, or where, for example, the patient speaks English as a second language, the clinician would likely rely more heavily on the objective refraction; however, the scenarios were constructed for patients with good VA and giving good, reliable subjective responses, as indicated at the beginning of the questionnaire.

### Limited cylinder power and axis techniques

The most common causes of prescription‐related complaints with new spectacles were cylinder changes (with oblique cylinders particularly implicated) at 38% of causes.^1^ An example of poor cylinder axis technique is shown in Question 6, where 39% (71/183) would make just one change to the axis from their starting prescription before prescribing that axis, and of these 71, 55 (30% of 183 respondents) would leave the axis at more than a 10° change for this broadly asymptomatic patient. There was no difference in approach with years of experience, with a mean of these 71 of 16 years, which was the same as all respondents. A few (*N* = 5, 3%) would make no changes at all to their starting prescription (i.e., making no cylinder power or axis assessment ), leaving the axis at the habitual prescription, which either shows marked adherence to the prescribing maxims discussed below or a surprising economy of effort.

Regarding cylinder power, further to the choice of cross‐cylinder used, the study addressed prescribing approaches. For the asymptomatic patients of Questions 7 and 8, 35% and 37%, respectively, would prescribe a full cylinder change, indicating a lack of consideration of the prescribing maxims—‘if it ain't broke don't fix it’ and ‘if it ain't broke don't fix it much’—and thus risking patient complaints with new spectacles. Interestingly, one third of rechecks could have been saved if these maxims had been applied.^1^

### Risk of over‐plusing

The risk of over‐plusing was investigated with Question 5. Of the 164 complete responses, 46 (28%) reported that they would start their subjective refraction with the objective result (a change of +0.50 DS) and look to prescribe it and just two suggested further checks (one reported use of a duochrome and one reported use of visual targets outside the test room). For a 35‐year‐old patient who reports no distance problems with their +3.00 DS spectacles but who has a little tiredness while reading, applying the ‘if it ain't broke don't fix it much’ maxim^4^, ^8^ and prescribing +3.25 DS would likely remedy the reading symptom without over‐correcting the distance. Prescribing a full +0.50 DS change risks over‐plusing the distance finding, particularly if the impact on threshold acuity and the optical length of a 6‐m test room (0.17 D) are not carefully considered. Of all respondents, just two suggested that they would consider threshold acuity or invite a patient to look out of a window with the proposed correction and just four mentioned that they would check with a duochrome. The high proportion of respondents who appear happy to prescribe a full plus prescription change is perhaps not surprising given that 91% (*N* = 185) reported having been taught to ‘push the plus’ or ‘MPMVA’^5^, ^6^, ^7^, ^8^ at university and perhaps suggests there was not sufficient emphasis on the risk of over‐plusing/under‐minusing when prescribing.^1^, ^8^

A risk of over‐plusing was mitigated by some (15% of 164 responses for Qu.5) by using a +0.50 DS confirmation lens and then changing the prescription each time by just +0.25 DS. This appears to be a good strategy (acknowledging personal bias, as indicated above) as the increased plus prescription changes err on the low side, and this approach could equally be applied to decreasing the minus spherical component of a prescription.

### Poor reading addition determination techniques

The visual tasks patients undertake at near could vary enormously, ranging from inspection of very fine detail at a close working distance to broad tasks undertaken at arm's length and these tasks performed by a range of abilities from a 40‐year‐old prodromal presbyope with good VA to an elderly patient with macular degeneration. With the decreasing range of clear vision associated with increasing addition power, it is necessary to confirm the patient's habitual reading distance and visual tasks and to check the range of clear vision with the proposed reading addition, either to provide for a range of movement either side of the desired task or to allow for tasks requiring different degrees of difficulty.^5^, ^6^, ^7^, ^8^ Question 11 was structured such that respondents had to volunteer that they would make these checks, with 32% not reporting that they would confirm the patient's habitual near working distance and 72% not reporting that they would check the range with the proposed addition. A difficulty of determination of near working distances with phoropters has been discussed^8^ and that 59% use one could have contributed to these low figures, but while some respondents might check distances in practice but did not report so here, these low figures are supported by the recheck study^1^ in which working distances were rarely recorded. With such small numbers of respondents fully checking near working distances, it is perhaps surprising that just 12% of recheck causes were due to the reading addition.^1^

### Reconciling prescription changes with symptoms and VA improvements

Question 10 represented a case often found in practice where an elderly patient with nuclear sclerotic cataracts undergoes a unilateral reduction in hyperopia, with an associated increase in anisometropia. The patient gives slightly vague responses associated with the cataract, and the clinician might over‐correct if care is not taken, reflected in the difference between the objective and the 0.25 DS further subjective change. With consideration of the presenting symptoms and improvements in VA from 6/15 to 6/7.5, it should be clear that a change of −2.00/−0.75 × 180 was excessive; however, 43% (*N* = 82) reported that they would prescribe this change without further checks, which is particularly concerning with an elderly patient. It might also be considered that the 24% (*N* = 44) who would prescribe the full objective change of Question 9 were also not reconciling changes. The increase of −0.75/−0.75, 10° towards the oblique, was designed to be slightly excessive for the improvement in VA; however, this is more marginal than the change in Question 10, and so it might be expected that far fewer respondents would look to prescribe the full change of Question 10. However, Question 10 provided a subjective prescription, and so it is likely some respondents would have been biased towards this compared with respondents to Question 9 where no subjective prescription was provided. These proportions for both Questions 9 and 10 (24% and 43%) could be greater than actual numbers in practice due to the straightforward question encouraging a quick response, rather than the conditional response questions that take the respondent through a process and so might elicit more consideration.

### Part refracting

An adaptation of the principle of part prescribing or prescription modification^3^, ^4^, ^8^, ^9^, ^10^ might be where a clinician stops subjective refraction tests at the point where the prescription will be issued, that is, not taking subjective tests to a full end‐point. While very few (*N* = 5–8) would not fully refract a plus sphere (Question 5), the cylinder axis (Question 6) or a small oblique cylinder (Question 8), 24% of 141 complete respondents would stop their subjective refraction of a 0.50 D cylinder change before a full end‐point (Question 7). This supports previous research^11^ and suggests that such a part‐refracting approach does appear to be used by a small number of optometrists in particular circumstances.

### Study limitations

We considered that if specific questions were asked regarding elements of technique, some respondents might be biased towards what they consider good practice rather than indicating their approaches in day‐to‐day practice.^12^ For example, regarding cylinder axis technique (Question 6), a question asking if respondents always bracket axes would likely have provided an overestimate of positive responses. Consequently, we endeavoured to take respondents through a process in order to determine the nuances of their technique. Question 11, regarding reading addition determination and prescribing, was provided as an open question for respondents to volunteer their technique. Had we specifically asked about working distance and range of clear vision measurement, many responses might have represented an impression of ‘good’ technique rather than techniques actually employed. As respondents were therefore left to volunteer their methods/approaches, our results could under‐represent good technique. However, this should be balanced with those who might describe a more comprehensive method than they actually use in practice, and consequently, our results are likely a reasonable estimate of actual practice.

The questions were constructed to allow for all types of individual approaches to refracting and prescribing; however, some respondents might have found some elements of the questions unclear depending on the nuance of their own approach.

## CONCLUSIONS

Many of the subjective refraction techniques reported in this study are poor and link to the recheck cases found in an earlier study.^1^, ^2^ The objective refraction, particularly that provided by an autorefractor, has an exaggerated influence on some optometrists, with 14% happy to prescribe full objective changes to asymptomatic patients and 15% full objective changes to patients with symptoms but a large objective change for the improvements in VA. Autorefractor users were significantly more likely to prescribe the objective prescription than retinoscope users (*χ* ^2^ = 16.8, *p* < 0.001).

The measurement of VA was found to be suboptimal by the vast majority of respondents, with 93% of respondents not measuring to a full threshold level. The use of test charts, which have a line smaller than 6/4.5 and recording acuities to full threshold levels, requires greater emphasis in CPD.

A mildly symptomatic patient with an objective change of +0.50 DS would have the full change prescribed by 28% (*N* = 46) of respondents, with the associated risk of over‐plusing and just two suggesting conducting any further checks. This was, however, balanced by the 15% of respondents who use a +0.50 DS confirmation lens and who then use +0.25 DS as an increment of plus increase.

Cross‐cylinder technique for cylinder determination appeared very economical to many, with 39% (*N* = 71) making just one change from their starting prescription before prescribing that result and 3% (*N* = 5) making no changes at all from their starting (habitual) prescription. When determining a reading addition, 32% would not check the habitual working distance for near tasks and 72% would not check the range of clear vision.

Presenting symptoms were often not reconciled with changes in prescription and improvements in VA, with 43% of respondents stating that they would prescribe a full change of −2.00 DS together with −0.75 DC for a VA improvement from 6/15 to 6/7.5 for an elderly patient.

## AUTHOR CONTRIBUTIONS

**Jeremy Beesley:** Conceptualization (lead); data curation (lead); formal analysis (lead); investigation (lead); methodology (equal); project administration (lead); resources (equal); validation (equal); visualization (lead); writing – original draft (lead); writing – review and editing (equal). **Christopher J. Davey:** Conceptualization (supporting); formal analysis (supporting); methodology (equal); project administration (supporting); supervision (supporting); visualization (supporting); writing – review and editing (supporting). **David B. Elliott:** Conceptualization (supporting); formal analysis (supporting); methodology (equal); project administration (supporting); supervision (equal); visualization (equal); writing – review and editing (equal).

## FUNDING INFORMATION

This study was not funded.

## CONFLICT OF INTEREST STATEMENT

The authors have no conflict of interest to declare.

## APPENDIX 1 Refraction methods questionnaire

**Refraction styles used by UK optometrists**

Jeremy Beesley

University of Bradford

Director, Beverley Specsavers

Dear Clinician,

Thank you for your time in completing this questionnaire. It aims to investigate the variety of styles and approaches that UK optometrists use in their day‐to‐day refractions. We expect a wide variety of styles given the variety of techniques taught by different supervisors in university clinics and by pre‐registration supervisors plus post‐qualification exchanges of ideas with colleagues and your own experiences of techniques that worked/didn't work with your patients.

We have designed a range of questions aimed at various elements of a refraction, and in completing the questionnaire, we would like you to imagine you have a patient in your room. All responses are anonymous. Please answer based on what you regularly do (and certainly not what you think might be expected).

If you have any questions regarding any aspect of this study, please do not hesitate to contact me at j.beesley2@bradford.ac.uk

Thank you again for your time.

All information is provided anonymously. Please do not give your name.

- How long have you been qualified (years)?

B Please select your gender.

**Table:**

Male	Female	Prefer not to say
		

C What type of practice do you work in? Please tick whichever most applies.

**Table:**

Independent	Multiple	Hospital	University eye clinic
			

D Are you employed or do you work as a locum?

**Table:**

Employed	Locum
	

E What refraction equipment do you routinely use? Please tick whichever you use.

**Table:**

Trial frame and lenses	Manual phoropter (Type?)	Digital phoropter (Type?)
		

F Which do you routinely use (i.e., for most patients)? Please tick.

**Table:**

Retinoscope	Autorefractor
	

When/with which type of patient would you use…?

Retinoscope…………………………………………………

Autorefractor………………………………………………

**The questions**

In the following scenarios, please assume the patient has good visual acuity (no pathology or amblyopia) and gives good/reliable subjective responses.

1. When determining the sphere powers of a prescription, which confirmation lenses do you routinely use (either confirmation lenses with a trial frame, or intervals of lens change with a phoropter)? Please tick which you usually use.

**Table:**

+0.25 and −0.25 DS	+0.50 and −0.25 DS	+0.50 and −0.50 DS
		

Other……………………………………………………………

2 When determining the cylinder axis and power, which cross‐cyl power would you routinely use?

**Table:**

±0.25 DC	±0.50 DC
	

Other……………………………………………………………

3 What is the bottom line you routinely use to measure visual acuities in young adults (20–30 years of age)? Please tick which applies.

**Table:**

6/6	6/5	6/4.5	6/3.8	Other (Give details)
				

4 At university were you taught, ‘Maximum plus for maximum visual acuity’, or ‘Push the plus’?

**Table:**

Yes	No	Cannot remember
		

5 You are refracting a 35‐year‐old patient, full‐time spectacles wearer, who reports good distance and near vision, but some tiredness when reading.

**Table:**

Habitual Rx	+3.00 DS ‘Bottom line^§^ VA’
Objective Rx (ret/autorefractor)	+3.50 DS R and L
(^§^Whichever you indicated in Qu 3.)	

With what Rx would you start subjective tests?

**Table:**

+3.00 DS	+3.25 DS	+3.50 DS
		

Dependent on your answer above, please answer (a) or (b) and then (c).

- *If you would start subjective tests with +3.00 DS, or +3.25 DS…*

If in Qu 1 you indicated you use a +0.25 DS and the patient answers ‘same’ for with and without the lens, do you…?

**Table:**

Change by +0.25 DS	Leave the sphere as it is
	

Other……………………………………………………………

If in Qu 1 you indicated you use a +0.50 DS and the patient answers ‘same’ for with and without the lens, do you…?

**Table:**

Change by +0.50 DS	Change by +0.25 DS	Leave the sphere as it is
		

Other……………………………………………………………

b *If you would start subjective tests with +3.50 DS* and with either the +0.25 DS or +0.50 DS (whichever you use) it is better without the lens and with −0.25 DS or −0.50 DS (whichever you use) it is ‘same’, would you…?

**Table:**

Reduce the sphere by 0.25 DS	Leave as it is (i.e., at +3.50 DS)
	

Other……………………………………………………………

c Would your approach have been different if you were checking the final sphere power (i.e., after using the cross cyl)? ……………………

6 You are refracting a broadly asymptomatic 35‐year‐old patient, but who suggests their ‘distance vision could be a bit better’.

**Table:**

Habitual (RE)	−0.50/−1.00 × 10	“Bottom line VA”‐2
Objective (RE, Ret/autorefractor)	−0.50/−1.00 × 30	

With what Rx would you start subjective tests (please tick)?

**Table:**

−0.50/−1.00 × 10 (Go to a)	−0.50/−1.00 × 30 (Go to b)	Other (please state) (Go to c)
		

- If you would start with −0.50/−1.00 × 10, checking the axis, the response of the cross‐cyl is towards 90, would you change the axis and if so, what to? …………
  If you changed it, the next response is ‘same’, would you leave the axis, or change to………… and repeat? …………
- If you would start with −0.50/−1.00 × 30, checking the axis, the response of the cross‐cyl is towards 180, would you change the axis and if so, what to? …………
  If you changed it, the next response is ‘same’, would you leave the axis, or change to………… and repeat? …………
- If you would start with ‘Other’, checking the axis, the response of the cross‐cyl is towards 90, would you change the axis and if so, what to? …………
  If you changed it, the next response is ‘same’, would you leave the axis, or change to………… and repeat? …………

7 You are refracting a 40‐year‐old *asymptomatic* patient.

**Table:**

Habitual (RE):	−1.00/−0.50 × 90	“Bottom line VA”.
Objective (RE, Ret/autorefractor):	−1.00/−1.00 × 90	“Bottom line VA”‐1

With what Rx would you start your subjective tests (please tick)?

**Table:**

−1.00/−0.50 × 90	−1.00/−0.75 × 90	−1.00/−1.00 × 90
		

Other……………………………………………………………

- If you started with −0.50 × 90, after sphere and axis checks the response to cross‐cyl power is better with −0.25 DC more, would you increase to −0.75 DC or keep at −0.50 DC because that is the largest cylinder you would prescribe anyway?

**Table:**

Keep at −0.50 DC	Increase to −0.75 DC
	

Other……………………………………………………………

b If you started with −0.50 × 90 and you did increase the power to −0.75 DC as above, or you started with −0.75 × 90, and the response is better with −0.25 DC more (i.e., to −1.00 DC), would you increase the cyl to −1.00 DC (and add +0.25 DS to compensate the change in cyl power) and check, or keep at −0.75 DC because that is the largest cylinder you would prescribe anyway?

**Table:**

Keep at −0.75 DC	Increase to −1.00 DC
	

Other……………………………………………………………

c If you started with −1.00 × 90 and the response with the cross‐cyl is ‘same’, would you leave the cyl at −1.00 DC or reduce to −0.75 DC?

**Table:**

Keep at −1.00 DC	Reduce to −0.75 DC
	

Other……………………………………………………………

8 You are refracting an *asymptomatic* 30‐year‐old patient.

**Table:**

Habitual (RE)	−1.00 DS	“Bottom line VA”
Objective (RE ret/autorefractor)	−1.00/−0.25 × 45	

Would you start your refraction with the cyl (i.e., −1.00/−0.25 × 45)?

**Table:**

Yes	(Go to a)
No	(Go to d)

- The cyl axis checks keep the cyl at 45, the power cross‐cyl is ‘same’, would you check with and without the cyl with best VA?

**Table:**

Yes	(Go to b)
No	(Go to c)

b The VA is just one or two letters better with the cyl, would you retain (i.e., prescribe) it?
  Yes
  No
c Would you retain (i.e., prescribe) the cyl?
  Yes
  No
d Would you use a cross‐cyl at 45/135 to check for a cyl?

**Table:**

Yes	(Go to e)
No	

e The answer is ‘not much difference’ (between 45 and 135), would you include the −0.25 × 45?
  Yes
  No

Any comments regarding Qu 8? …………………………………………………………………

…………………………………………………………………

9 A 22‐year‐old patient reports slightly blurred distance vision, RE;

**Table:**

Habitual (RE):	−1.00/−0.50 × 35	6/9 + 2
Objective (RE, Ret/autorefractor):	−1.75/−1.25 × 45	“Bottom line VA”

- Given the symptoms, habitual Rx and VA, what final prescription might you expect to prescribe? ………………………………………………………………
- With what Rx would you start your subjective tests? ………………………………………………………………
  Why? ………………………………………………………

10 A 72‐year‐old patient with (nuclear sclerotic) cataract reports problems with distance vision in their varifocals. The patient's responses are slightly vague.

**Table:**

Habitual	R + 3.00/−0.50 × 180 6/15 + 2.50 Add N5
	L + 3.00 DS 6/9 + 2 + 2.50 Add N5

Objective (Ret/autorefractor)	R + 1.25/−1.25 × 180
	L +2.75 DS

Subjective:	R +1.00/−1.25 × 180 6/7.5 + 3.00 Add N5
	L +2.75 DS 6/7.5 + 3.00 Add N5

Would you prescribe this subjective Rx, or what tests or checks would you use before prescribing? …………………………………………………………………

11 You are refracting a 53‐year‐old patient who reports tiredness when reading without their (single‐vision distance) glasses. There is no change with their distance prescription (−0.50 DS R and L). Describe what you *do and say* to the patient when determining the reading Addition.

…………………………………………………………………………………………
…………………………………………………………………………………………
…………………………………………………………………………………………
…………………………………………………………………………………………
…………………………………………………………………………………………
………………………………………………………………
…………………………………………………………………………………………
………………………………………………………………………………

12 Do you have any comments regarding your routine refraction methods and any element of this questionnaire?
  …………………………………………………………………………………………
  …………………………………………………………………………………………
  ………………………………………………………………………
  Thank you very much for your time in completing this questionnaire.

## APPENDIX 2 Potential errors of refraction with a ±0.50 D cross‐cylinder

If a patient has in the trial frame a prescription of, for example, −3.00 DC, but an ideal end refraction of −3.25 DC, use of a ±0.25 D cross‐cylinder will give (i) the ideal −3.25 DC and (ii) the worse prescription of −2.75 DC, and therefore, the patient response is likely to be in definite favour of (i). If, however, a ±0.50 D cross‐cylinder is used, (i) becomes −3.50 DC, which is not ideal, and (ii) −2.50 DC. (i) is better, but the patient is being asked to compare two prescriptions, neither of which is ideal, and therefore possibly a difficult judgement to make, with an associated potential error of interpretation of the response.

Regarding cylinder axis, the same reasoning applies; however, options (i) and (ii) are more blurred either side of the ideal with a ±0.50 D cross‐cylinder and consequently a harder judgement for the patient to make compared with the use of a ±0.25 cross‐cylinder.
